# Cervical Human Papillomavirus Infection (HPV) and High Oncogenic Risk Genotypes among Women Living with HIV in Asia: A Meta-Analysis

**DOI:** 10.3390/jcm10091911

**Published:** 2021-04-28

**Authors:** Florian Verrier, Sophie Le Coeur, Tristan Delory

**Affiliations:** 1 Faculty of Associated Medical Sciences, Chiang Mai University, Chiang Mai 50200, Thailand; lecoeur@ined.fr (S.L.C.); delory.tristan@gmail.com (T.D.); 2 Institut National d’Etudes Démographiques (INED), 9 Cours des Humanités, 93322 Aubervilliers, France; 3 Institut de Recherche Pour le Développement (IRD) UMI 174-PHPT, 13002 Marseille, France; 4 Délégation à la Recherche Clinique et à l’Innovation (DRCI), Centre Hospitalier Annecy-Genevois, 74370 Epargny Metz-Tessy, France

**Keywords:** women, HIV, HPV, oncogenic HPV, Asia, systematic review, meta-analysis, prevalence

## Abstract

Women living with HIV (WLHIV) are prone to harbor several high-risk human papillomavirus (HR-HPV) genotypes and to develop cervical cancerous lesions. Data on HPV prevalence in these women are needed to inform immunization programs, especially in Asia where few data are available. We conducted a systematic review and meta-analysis to estimate the prevalence of HPV and HR-HPV cervical infection in WLHIV in Asia and identify possible sources of heterogeneity for HR-HPV carriage. Pooled prevalence and its 95% confidence interval (95CI) were estimated using the inverse-variance weighting method. Linear regression weighted on study size was used to identify sources of heterogeneity. Among 7834 WLHIV (40 studies), the prevalence of HPV infection was 42.6% (95CI, 38.2% to 47.1%), and 34.6% (95CI, 30.3% to 39.1%) harbored HR-HPV genotypes, with significant heterogeneity across countries. In India, Thailand, and China, HPV-16 was the most frequent genotype (10.3%), followed by HPV-52 (5.4%), HPV-58 (5.0%), HPV-18 (4.1%), and HPV-33 (3.3%). In these women, most of whom were receiving antiretroviral therapy, we did not identify determinants of heterogeneity for HR-HPV infection. Our results underline the need for immunization programs based on nonavalent or new generation vaccines to prevent cervical cancer in WLHIV in Asia.

## 1. Introduction

Human papillomavirus (HPV) infection is the main determinant of cervical cancer in women [1]. Nearly 70% of cervical cancers worldwide are related to high oncogenic risk (HR)-HPV genotypes 16 and 18, but at least 11 other HR-HPV types are also known to be associated with cervical cancer [2,3]. The distribution of HR-HPV genotypes varies by geographic area, demographics, and sexual risk behaviors [4,5,6]. Most HPV infections are cleared spontaneously but may persist and induce the development of precancerous and cancerous lesions [7]. Recent data showed that the cervicovaginal microenvironment, involving a complex interaction between microbiota, HPV infection, and inflammation, influences cervical carcinogenesis [8].

According to the World Health Organization (WHO), Asia has the highest incidence rate of cervical cancer after sub-Saharan Africa. Each year, 300,000 new cervical cancers are diagnosed among Asian women, and 150,000 die from cervical cancer [9]. Depending on the subregion, the incidence rate of cervical cancers ranges from 4.1 per 100,000 women in Western Asia to 17.8 per 100,000 women in Southeast Asia [9]. Recent estimates indicate a future increase in the burden of cervical cancer, concentrated in Africa and Asia [10].

Human immunodeficiency virus (HIV) infection is also a strong determinant of cervical cancer [11]. The proportion of new cervical cancer attributable to HIV is up to 1.2% in Southeast Asia and Europe and can be as high as 21% in sub-Saharan Africa [11]. Compared to women from the general population, those living with HIV are at higher risk of incident and persistent HPV infection [12,13]. Furthermore, when infected, women living with HIV (WLHIV) are more likely to harbor several oncogenic genotypes and are thereby prone to develop cervical precancerous or cancerous lesions [14,15,16,17,18].

Over the last two decades, the scaling-up of antiretroviral therapy (ART) has dramatically increased the life expectancy of WLHIV [19,20,21], leading to higher incidence and prevalence of cervical cancers related to HR-HPV [22].

A global meta-analysis was carried out by Clifford et al. to estimate the worldwide prevalence of HPV and HR-HPV in WLHIV, but data on Asia were limited [4].

In our meta-analysis, we estimate the prevalence of HPV and HR-HPV cervical infection in WLHIV in Asia and identify possible sources of heterogeneity for HR-HPV carriage.

## 2. Materials and Methods

This systematic review was designed according to the Preferred Reporting Items for Systematics Reviews and Meta-Analyses (PRISMA) statement [23,24].

### 2.1. Study Selection

We focused our systematic review on the Asian continent, including countries of the Middle East (Asia–Africa boundary). Source material was obtained from peer-reviewed published literature, limited to English and French, using the following key search terms and connectors: (human immunodeficiency virus or AIDS) AND (HPV or human papillomavirus) AND (gynecological or cervix or cervical) AND (asia or asian or afghanistan or armenia or azerbaijan or bahrain or bangladesh or bhutan or brunei or cambodia or china or cyprus or georgia or india or indian or indonesia or iran or iraq or israel or japan or jordan or kazakhstan or korea or kuwait or kyrgyzstan or laos or lebanon or malaysia or maldives or mongolia or myanmar or nepal or oman or pakistan or philippines or qatar or russia or saudi arabia or singapore or sri lanka or syria or taiwan or tajikistan or thailand or timor or turkey or turkmenistan or united arab emirates or uzbekistan or vietnam or yemen). The search was conducted in the Medline database using either MeSH headings or free-text terms. The literature was last searched in October 2020, targeting the last 30 years.

Titles and abstracts of the articles were assessed. Articles were excluded if (1) the language was not English or French; (2) the study did not take place in Asia; (3) subjects were not women; (4) subjects were women not living with HIV; (5) the localization of HPV infection was not the cervix; and (6) HPV prevalence (overall or by genotype) was not provided. Meta-analyses and literature reviews were also excluded. When abstracts were unavailable or failed to provide sufficient information, full articles were retrieved to assess eligibility criteria. Furthermore, to avoid duplicates, if data subsets originating from a single study were published in more than one article with redundant information, only the publication with the largest sample size was included in the meta-analysis.

### 2.2. Data Extraction

For each study, the following information was extracted:

- Study settings: study identification (first author, publication year, journal of publication); location (country, region, or city) where the study was performed.
- Sociodemographic data of WLHIV: number of WLHIV enrolled; mean age of WLHIV; and percentage of WLHIV who: use condoms consistently (always or most of the time), have at least a primary education, smoke, are sex workers, and have had at least one pregnancy.
- Data related to HPV infection: number of WLHIV tested for HPV infection; HPV test used; number of HPV genotypes tested; percentage of WLHIV with HPV infection and multiple HPV infection, and mean number of genotypes for those with multiple infection; percentage of WLHIV with any HR-HPV infection; percentage of WLHIV positive for each of the 13 HR-HPV genotypes, according to WHO’s classification [3].
- Data related to HIV infection: mean CD4 cell-count nadir and when tested for HPV; mean HIV-RNA viral load and percentage of WLHIV with detectable viral load when tested for HPV; percentage of WLHIV receiving antiretroviral therapy, and mean duration since treatment initiation.

The extracted data were cross-checked by two independent reviewers (FV and TD). A third expert was consulted if needed (SL). When mean was not provided, we used the median and the interquartile range to compute the mean and the standard deviation [25].

### 2.3. Statistical Analyses

Within each study, we estimated the prevalence of HPV infection as the number of WLHIV harboring any HPV genotype, divided by the total number of women who underwent HPV testing. The prevalence of HR-HPV infection overall and by oncogenic genotype was calculated as the number of WLHIV harboring any of the 13 oncogenic genotypes, divided by the total number of women who underwent HPV testing. The 13 oncogenic genotypes (16, 18, 31, 33, 35, 39, 45, 51, 52, 56, 58, 59, 68) were considered based on the WHO classification [3].

In the meta-analysis, we estimated the pooled prevalence and its 95% confidence interval (95CI) using the inverse-variance weighting method for HPV infection, HR-HPV genotypes combined, and for each HR-HPV genotype. The analysis of pooled prevalence of HR-HPV was restricted to countries with at least three studies and more than 100 WLHIV, in which women were tested for the 13 HR-HPV genotypes. We tabulated the prevalence of HPV and HR-HPV infection across countries using Forest plots. We compared the pooled prevalence within countries using the chi-squared test. We used a random-effects model to reflect the differential scaling of antiretroviral therapy programs throughout Asia [26]. Heterogeneity was measured using Cochrane’s *Q* test. We used a linear regression weighted on study size to estimate the strength of association between HR-HPV infection and potentially confounding, random effects: mean age; mean CD4 at time of HPV testing; use of antiretroviral therapy (yes/no); undetectable viral load (yes/no); consistent condom use (yes/no); educational level (at least primary school vs. no school); smoking (yes/no); sex workers (yes/no); parity history. Associations are reported using exponential of β coefficients (Exp(β)) with their 95CI.

A sensitivity analysis was performed to estimate pooled prevalence of HR-HPV genotypes, including all studies providing prevalence for genus alpha genotypes: genus alpha-5 group (HPV-18, 45, 97, 59, 68, 39, 70, 85), alpha-6 group (HPV-82, 51, 30, 53, 56, 66), alpha-7 group (HPV-26, 69), alpha-9 group (HPV-16, 31, 35, 33, 58, 52, 67) and alpha-11 group (HPV-34, 73).

All tests were two-tailed, and a *p* value of ≤0.05 was considered statistically significant. Analyses were conducted with the R software environment (version 3.6.1) using the Meta package.

## 3. Results

### 3.1. Studies Included

**Figure 1:**
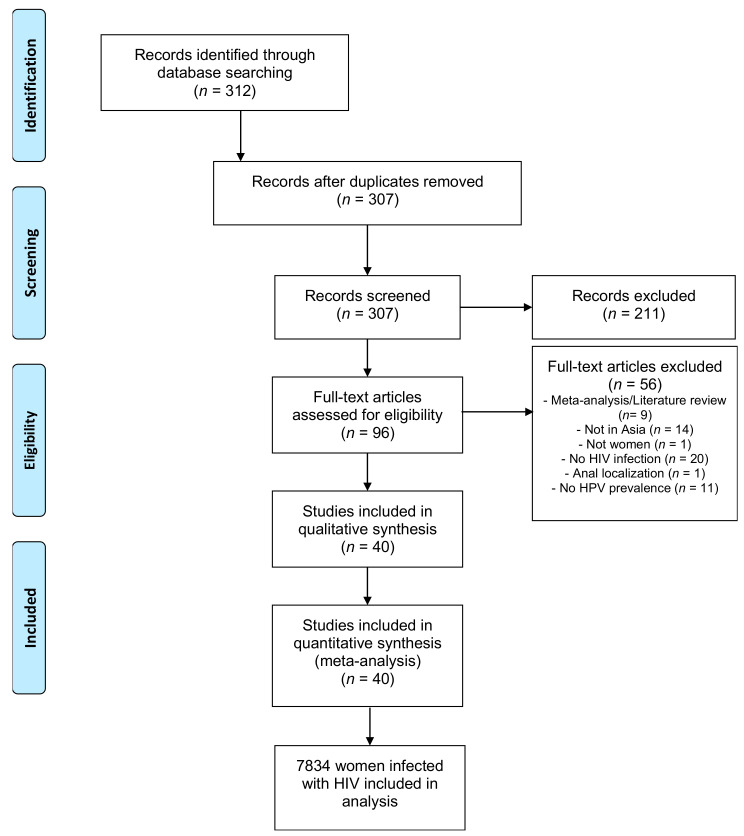
Process of selecting studies used in the meta-analysis.

Systematic review identified 312 records. After screening titles and abstracts, we excluded 216 articles, including five duplicates. Of the remaining 96, 40 were eligible and were included in the meta-analysis. Figure 1 details the systematic process for article selection.

Among these 40 studies (7834 WLHIV), 15 were from India, 9 from Thailand, 6 from China, 2 from Cambodia, Taiwan, and Iran; only one was available from South Korea, Vietnam, Oman, and Israel.

Table 1 presents the characteristics of the 7834 WLHIV included in the analysis. The pooled mean age was 32 years (27 studies). About one-quarter of women were smokers (15 studies, 24.1%), and one-fifth were reported to be sex workers (13 studies, 17.1%). Half of them used condoms consistently (12 studies, 54.3%), and most had been pregnant at least once (14 studies, 83.9%). The proportion of women having attended primary school was 72.9% (17 studies). Half of women were under antiretroviral therapy (31 studies, 54.6%), and when reported, half of them had an undetectable HIV-RNA viral load (nine studies, 52.6%). The pooled mean CD4 cell count when tested for HPV was 422 cells/mm^3^ (14 studies).

### 3.2. HPV Prevalence

**Figure 2:**
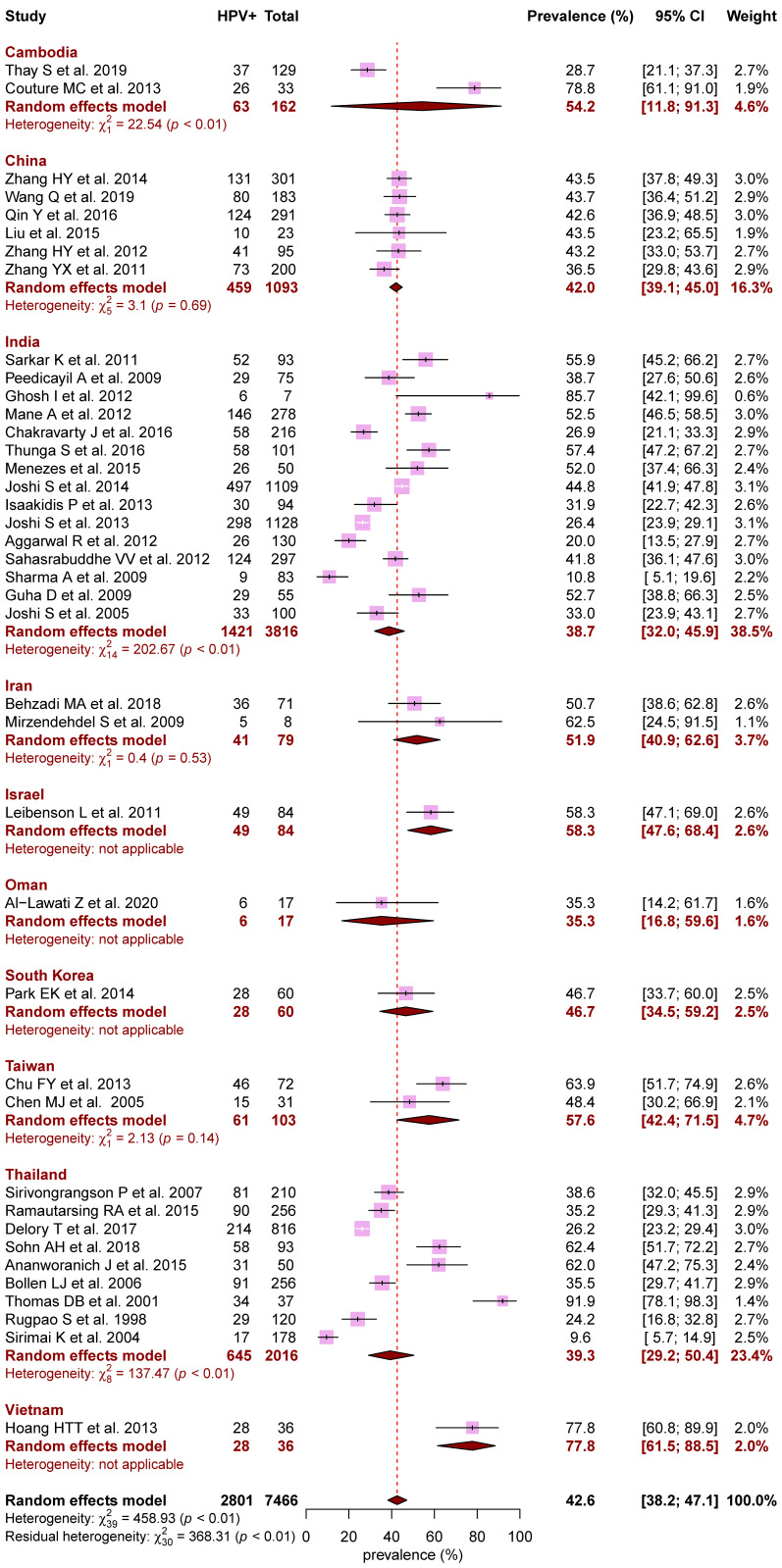
Forest plot of HPV prevalence across countries in Asia. Meta-analyses with random-effects models. HPV: human papillomavirus; CI: confidence interval.

Figure 2 presents the pooled prevalence of HPV, overall and by country. Among the 7466 WLHIV tested, the overall prevalence for HPV infection was 42.6% (95CI, 38.2% to 47.1%) with significant heterogeneity across countries and within countries for India, Cambodia, and Thailand (*p* < 0.01). The overall prevalence of multiple HPV infection was 15.8% (95CI, 12.2% to 20.2%; 17 studies).

Among 6925 women living in countries where at least three studies had been carried out, the estimates of prevalence ranged from 38.7% (95CI, 32.0% to 45.9%) in India (15 studies) to 39.3% (95CI, 29.2% to 50.4%) in Thailand (nine studies), and 42.0% (95CI, 39.1% to 45.0%) in China (six studies) (*p* < 0.01).

### 3.3. HR-HPV Prevalence and Distribution of HR-HPV Genotypes

**Figure 3:**
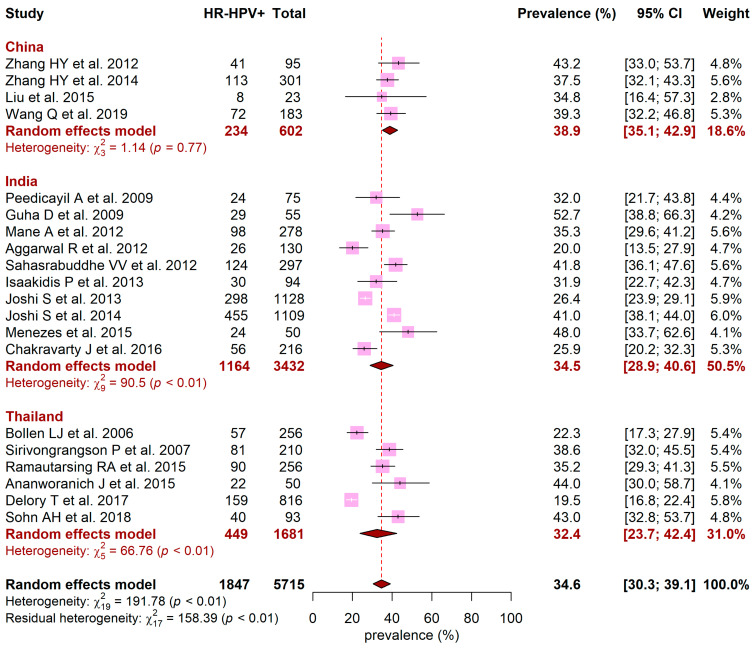
Forest plot of HR-HPV prevalence across China, India, and Thailand. Meta-analyses with random-effects models. HR-HPV: high-risk human papillomavirus; CI: confidence interval.

Figure 3 presents the pooled prevalence of HR-HPV for India, Thailand, and China. Among 5715 women, the overall HR-HPV prevalence in these countries was 34.6% (95CI, 30.3% to 39.1%) and was heterogeneous between countries, ranging from 32.4% in Thailand (95CI, 23.7% to 42.4%) to 34.5% in India (95CI, 28.9% to 40.6%) and 38.9% in China (95CI, 35.1% to 42.9%) (*p* < 0.01). Except for China, significant heterogeneity was observed in India and Thailand (*p* < 0.01). Results were similar in the sensitivity analysis including all studies providing prevalence for genus alpha genotypes (Supplementary Figure S1).

**Figure 4:**
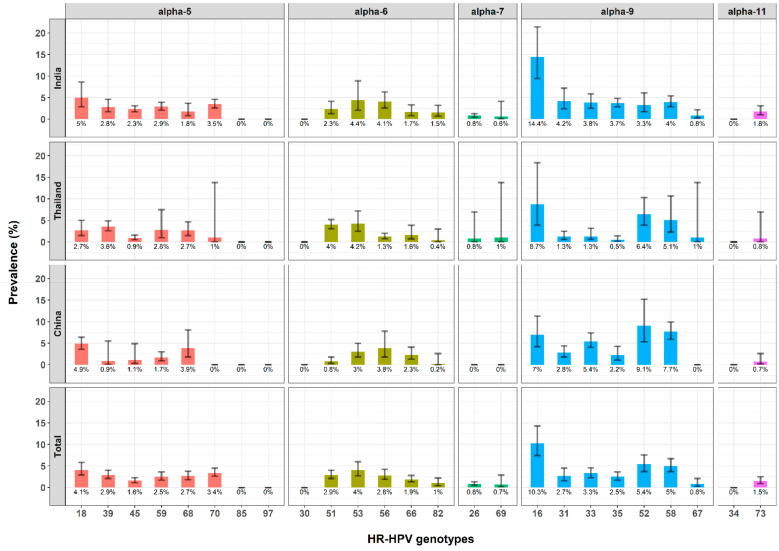
Prevalence of HR-HPV genotypes by country (China, India, and Thailand) and genus alpha group. HR-HPV: high-risk human papillomavirus. Each bar indicates the prevalence of the HPV genotype and its 95% confidence interval. Red bars for genus alpha-5 group, green bars for genus alpha-6, emerald green bars for genus alpha-7, blue bars for genus alpha-9, and purple bars for genus alpha-11.

Figure 4 summarizes the prevalence of each HR-HPV genotype within India, Thailand, and China, and within these three countries combined, by genus alpha group. Overall, HPV genotypes of the genus alpha-9 group were the most prevalent. HPV-16 was the most frequent genotype at 10.3% (95CI, 7.4% to 14.3%), followed by HPV-52 at 5.4% (95CI, 3.7% to 7.6%), HPV-58 at 5.0% (95CI, 3.7% to 6.7%), HPV-18 at 4.1% (95CI, 2.9% to 5.8%), and HPV-33 at 3.3% (95CI, 2.3% to 4.6%). In China, four genotypes had prevalence beyond 5%: HPV-52, HPV-58, HPV-16, and HPV-33. Three genotypes had prevalence beyond 5% in Thailand (HVP-16, HPV-52, and HPV-58), and two in India (HPV-16 and HPV-18).

### 3.4. Sources of Heterogeneity for HR-HPV Prevalence

Table 2 shows the results of the univariate weighted regression conducted to assess the sources of heterogeneity for HR-HPV prevalence among women in India, Thailand, and China. In this population including two-thirds (66.3%) of women under antiretroviral therapy and with good immunological response (mean CD4 at 449 cells/mm^3^), no factor was significantly associated with higher prevalence of HR-HPV infection. 

## 4. Discussion

Our meta-analysis reveals that 42.6% of the WLHIV in Asia were infected with HPV, and 34.6% harbored oncogenic HPV genotypes. In India, Thailand, and China, countries for which information on HR-HPV genotypes was available, HPV-16 was the most frequent genotype (10.3%), followed by HPV-52 (5.4%), HPV-58 (5.0%), HPV-18 (4.1%), and HPV-33 (3.3%). In these women, most of whom were receiving antiretroviral therapy and with a good immunological and virological response, we did not identify any determinant of heterogeneity for HR-HPV infection.

In a previous global meta-analysis, the prevalence of HPV among WLHIV was estimated at 33% across nine studies in Asia, varying by cytological finding from 25% in those with normal findings, to 76% in those with atypical squamous cells of undetermined significance or higher grade lesions (ASCUS+) [27]. The differences with the prevalence estimated from our meta-analysis may be explained by women’s profile, cytological findings, coverage of antiretroviral therapy, and tests used for HPV genotyping. 

Compared to previous meta-analyses describing HPV prevalence among HIV negative women, our results show that WLHIV in Asia have a 1.4 (IC95 1.3–1.4) to 6.7 (IC95 6.6–6.8) increased risk of being infected with HPV, depending on population characteristics [28,29,30,31].

In our meta-analysis restricted to India, Thailand, and China, HR-HPV prevalence was consistent at around 34.6%, and the distribution of genotypes was similar to that previously reported, with the highest rate for HPV-16 and HPV-52 infections [4]. In China, the three most prevalent genotypes were HPV-52, HPV-58, and HPV-16, similar to recent reports among the general population [32,33].

We could not confirm the role of known factors associated with a higher prevalence of HR-HPV, such as age and educational level, or of factors related to HIV infection (antiretroviral intake and immune status). However, most women (62.6%) had an undetectable viral load and a good immunological status (mean CD4 449 cells/mm^3^), suggesting that HIV treatment reduces the risk of HR-HPV infection [34]. Moreover, to control for biased estimation in HR-HPV prevalence, we excluded countries for which only small data sets were available: Cambodia, Israel, Oman, South Korea, Taiwan, and Vietnam. We also restricted our analysis to studies for which only the 13 oncogenic genotypes were considered, which may have reduced our ability to identify sources of heterogeneity. Furthermore, at least half of the studies had missing data for demographics, behaviors, or factors related to HIV infection, resulting in a risk of aggregation bias [35].

We could not provide the distribution of genotypes by cytological or histological cervical findings since this information was documented in only a few studies. Similarly, phylogenetic analysis was not possible. Complete individual-level data linking HPV genotyping to the presence of cervical lesions would be needed to address these issues. Additional measurement biases may have occurred, as the performance in the detection of (oncogenic) HPV varies according to the test used. Incoming technologies for HPV DNA testing, including circulating HPV DNA (ct-DNA), could improve the prevalence estimations and risk stratification [36].

A recent study demonstrated an increased risk of cervical cancer among WLHIV, compared to uninfected women [11], underlining the importance of cervical cancer screening and HPV vaccination in WLHIV. Nevertheless, our results provide valuable information about the HPV genotypes to be targeted in immunization programs for WLHIV in Asia. However, to reach the projected impact of HPV vaccination and optimize its benefits, several barriers need to be overcome. It will require strong political support for access to affordable (free) vaccines, improved awareness among health care workers, and enhanced individual or parental acceptance [10,37,38].

## 5. Conclusions

In Asia, the scaling-up of antiretroviral therapy programs seems to be effective for protecting WLHIV from oncogenic HPV infection. HPV-16 was the most frequent genotype, but closely followed by HPV-52, -58, -18, and -33, which underlines the need for immunization programs based on nonavalent or new generation vaccines to fight cervical cancer in these women [39].

## Figures and Tables

**Table 1:** Pooled characteristics of the overall population.

Characteristics	Number of Studies	Total Number of WLHIV	Number of WLHIV with the Characteristic	Pooled Mean or Proportion	95CI
Age (years)	27	5683	-	32 years	(29 to 35)
Under ART (%)	31	7383	4574	54.6%	(44.2% to 64.5%)
Undetectable viral load (%)	9	1989	1248	52.6%	(31.7% to 72.7%)
CD4 when tested for HPV (cell/mm^3^)	14	3053	-	422 cells/mm^3^	(361.7 to 482.3)
Smokers (%)	15	4443	850	24.1%	(17.9% to 31.6%)
Sex workers (%)	13	1630	254	17.1%	(10.1% to 27.5%)
Consistent condom use (%)	12	2406	1290	54.3%	(40.6% to 67.3%)
At least one pregnancy (%)	14	4686	4012	83.9%	(73.5% to 90.7%)
At least primary education (%)	17	5664	4142	72.9%	(60.7% to 82.4%)

WLHIV: women living with HIV; HPV: human papillomavirus; CI: confidence interval; ART: antiretroviral therapy; Consistent condom use: always or most of the time.

**Table 2:** Associations between HR-HPV prevalence and study characteristics.

Characteristics	Number of Studies	Total WLHIV in the Studies	Number of WLHIV with HR-HPV Results	Pooled Mean or Proportion (95CI)	Univariate Analysis		
					Exp(β)	95CI	*p* Value
Age (years) per year increment	16	4363		32 (28–36)	0.99	[0.99–1.00]	0.052
Under ART	19	5756	3958	66.3% (56.2–75%)	0.93	[0.81–1.07]	0.349
Undetectable viral load	6	1667	1163	62.6% (36.5–83%)	1.06	[0.77–1.45]	0.736
CD4 when tested for HPV (cells/mm^3^)	10	2353		448.6 (388.4–508.8)	1.00	[0.99–1.00]	0.731
Smokers	9	3449	610	21.5% (15.8–28.6%)	1.12	[0.92–1.34]	0.332
Sex workers	4	772	76	8.9% (5–15.5%)	0.99	[0.10–9.43]	0.993
Consistent condom use	8	1892	1130	61.3% (48.5–72.7%)	1.26	[0.89–1.77]	0.243
At least one pregnancy	9	3942	3375	82.5% (66.3–91.9%)	0.95	[0.73–1.25]	0.741
At least primary education	12	4785	3716	78.2% (68.1–85.7%)	1,17	[0.87–1.59]	0.326

WLHIV: women living with HIV; HR-HPV: high-risk human papillomavirus; CI: confidence interval; ART: antiretroviral therapy; Consistent condom use: always or most of the time.

## Data Availability

Data supporting this research have been extracted from published literature.

## Supplementary Materials

The following are available online at https://www.mdpi.com/article/10.3390/jcm10091911/s1, Figure S1: Forest plot of HR-HPV prevalence across China, India, and Thailand. Sensitivity analysis.
