# Supplementary material for: Cervical Human Papillomavirus Infection (HPV) and High Oncogenic Risk Genotypes among Women Living with HIV in Asia: A Meta-Analysis
Source: J Clin Med. 2021 Apr 28;10(9):1911. doi: 10.3390/jcm10091911 (PMC8125216; doi:10.3390/jcm10091911)
Supplement: Supplementary file 1 [file jcm-10-01911-s001.zip › jcm-1187683-supplementary/jcm-1187683-supplementary.pdf]

Supplementary Material

Cervical human papillomavirus infection (HPV) and high-oncogenic risk genotypes among women living with HIV in Asia: a meta-analysis

Study	HR-HPV+	Total	Prevalence (%)	95% CI	Weight
**China**					
Zhang YX et al. 2011	67	200	33.5	[27.0; 40.5]	4.1%
Zhang HY et al. 2012	41	95	43.2	[33.0; 53.7]	3.7%
Zhang HY et al. 2014	113	301	37.5	[32.1; 43.3]	4.3%
Liu et al. 2015	8	23	34.8	[16.4; 57.3]	2.2%
Qin Y et al. 2016	124	291	42.6	[36.9; 48.5]	4.3%
Wang Q et al. 2019	72	183	39.3	[32.2; 46.8]	4.1%
**Random effects model**	**425**	**1093**	**38.9**	**[36.0; 42.0]**	**22.7%**
Heterogeneity: $\chi^2_5 = 5.26$ ($p = 0.38$)					
**India**					
Joshi S et al. 2005	33	100	33.0	[23.9; 43.1]	3.7%
Peedicayil A et al. 2009	24	75	32.0	[21.7; 43.8]	3.4%
Guha D et al. 2009	29	55	52.7	[38.8; 66.3]	3.3%
Sarkar K et al. 2011	43	93	46.2	[35.8; 56.9]	3.7%
Mane A et al. 2012	98	278	35.3	[29.6; 41.2]	4.3%
Aggarwal R et al. 2012	26	130	20.0	[13.5; 27.9]	3.6%
Sahasrabuddhe VV et al. 2012	124	297	41.8	[36.1; 47.6]	4.3%
Isaakidis P et al. 2013	30	94	31.9	[22.7; 42.3]	3.6%
Joshi S et al. 2013	298	1128	26.4	[23.9; 29.1]	4.5%
Joshi S et al. 2014	455	1109	41.0	[38.1; 44.0]	4.6%
Menezes et al. 2015	24	50	48.0	[33.7; 62.6]	3.2%
Chakravarty J et al. 2016	56	216	25.9	[20.2; 32.3]	4.1%
Thunga S et al. 2016	54	101	53.5	[43.3; 63.5]	3.8%
**Random effects model**	**1294**	**3726**	**36.6**	**[31.3; 42.1]**	**50.0%**
Heterogeneity: $\chi^2_{12} = 110.7$ ($p < 0.01$)					
**Thailand**					
Rugpao S et al. 1998	19	120	15.8	[9.8; 23.6]	3.4%
Bollen LJ et al. 2006	57	256	22.3	[17.3; 27.9]	4.1%
Sirivongrangson P et al. 2007	81	210	38.6	[32.0; 45.5]	4.2%
Ramautarsing RA et al. 2015	90	256	35.2	[29.3; 41.3]	4.2%
Ananworanich J et al. 2015	22	50	44.0	[30.0; 58.7]	3.2%
Delory T et al. 2017	159	816	19.5	[16.8; 22.4]	4.5%
Sohn AH et al. 2018	40	93	43.0	[32.8; 53.7]	3.7%
**Random effects model**	**468**	**1801**	**29.8**	**[22.0; 38.9]**	**27.2%**
Heterogeneity: $\chi^2_6 = 74.28$ ($p < 0.01$)					
**Random effects model**	**2187**	**6620**	**35.1**	**[31.2; 39.2]**	**100.0%**
Heterogeneity: $\chi^2_{25} = 242.07$ ($p < 0.01$)					
Residual heterogeneity: $\chi^2_{23} = 190.24$ ($p < 0.01$)					

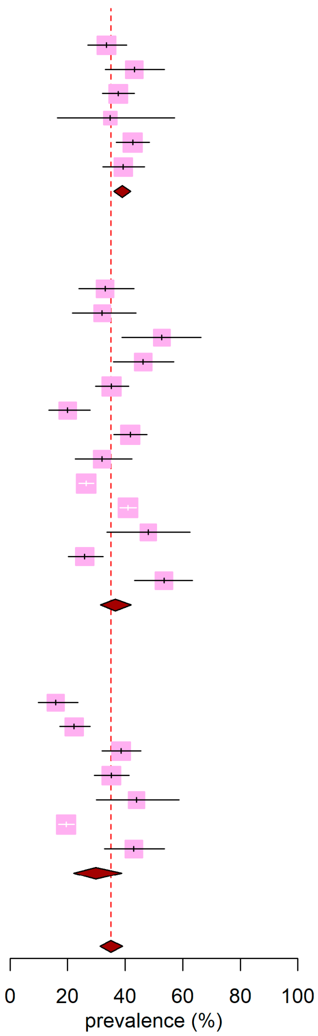

Figure S1. Forest plot of HR-HPV prevalence across China, India, and Thailand. Sensitivity analysis. Meta-analyses with random-effects models. HR-HPV: high-risk human papillomavirus; CI: confidence interval.
